# Prognostic Factors and Overall Survival After Pericardiocentesis in Patients With Cancer and Thrombocytopenia

**DOI:** 10.3389/fcvm.2021.638943

**Published:** 2021-04-21

**Authors:** Nathaniel R. Wilson, Michelle T. Lee, Clarence D. Gill, Astrid Serauto Canache, Teodora Donisan, Dinu V. Balanescu, Juhee Song, Nicolas Palaskas, Juan Lopez-Mattei, Mehmet Cilingiroglu, Konstantinos Marmagkiolis, Cezar A. Iliescu

**Affiliations:** ^1^Division of Cardiology, Department of Internal Medicine, The University of Texas Health Science Center at Houston McGovern Medical School, Houston, TX, United States; ^2^Division of Cardiology, Department of Internal Medicine, Baylor College of Medicine, Houston, TX, United States; ^3^Division of Cardiology, Department of Internal Medicine, The University of Texas MD Anderson Cancer Center, Houston, TX, United States; ^4^Division of Biostatistics, University of Texas MD Anderson Cancer Center, Houston, TX, United States

**Keywords:** pericardial effusion, pericardiocentesis, thrombocytopenia, safety, cancer

## Abstract

**Background:** Pericardiocentesis is an important diagnostic and therapeutic tool for cancer-associated pericardial effusion. Limited safety and outcomes data exists regarding the management of malignancy-related pericardial effusion in patients with thrombocytopenia.

**Objectives:** Our study aimed to analyze prognostic factors and overall survival (OS) after pericardiocentesis in thrombocytopenic cancer patients.

**Methods and Results:** A retrospective review of 136 thrombocytopenic cancer patients who underwent primary percutaneous pericardiocentesis was performed. Degree of thrombocytopenia was classified by platelet count recorded on day of pericardiocentesis: 75–149 × 10^3^ cells/μL (41%); 50–74 × 10^3^ cells/μL (10%); 25–49 × 10^3^ cells/μL (24%); <25 × 10^3^ cells/μL (25%). Median OS was 2.6 months and median follow-up was 37.4 months. Kaplan-Meier survival analysis showed significant OS differences among thrombocytopenia severity groups (*p* = 0.023), and worse OS with platelets <100 vs. ≥100 × 10^3^ cells/μL (*p* = 0.031). By univariate analysis, thrombocytopenia severity was associated with increased risk of death (HR 0.993; 95% CI 0.989–0.997; *p* = 0.002). Poor prognostic factors for OS were advanced cancer, malignant effusion, elevated international normalized ratio (INR), quantity of platelet transfusions, and platelet transfusion resistance. However, thrombocytopenia severity became insignificant for OS (*p* = 0.802), after adjusting for advanced cancer and INR.

**Conclusions:** For patients with malignancy-related large pericardial effusion and thrombocytopenia, pericardiocentesis is a feasible intervention and should be considered due to low complication rates. There is no absolute contraindication to pericardiocentesis in case of hemodynamic instability, even with severe thrombocytopenia.

## Introduction

Cancer causes pericardial disease by direct structural infiltration or indirectly via chemotherapy, radiotherapy, immunotherapy, or opportunistic infections (1, 2). Pericardial effusion (PE) is associated with malignancy in up to 20% of cases in autopsy studies, and 33% of patients with symptomatic PE have concomitant cancer in a large retrospective review (3–5). Pericardiocentesis is an important diagnostic and therapeutic tool for the cardio-oncologist as large PE manifesting with tachycardia, dyspnea, chest pain, cardiac tamponade, or cardiogenic shock are common (6). According to the European Society of Cardiology 2015 guidelines, in cardiac tamponade with underlying malignancy requiring therapeutic pericardiocentesis, extended pericardial drainage is indicated (class IB level recommendation) (7).

Limited data and safety outcomes exist regarding the management of malignancy-related PE in patients with thrombocytopenia (platelet count <150 × 10^3^ cells/μL). In thrombocytopenic patients, platelet count has an imprecise association with increased risk of bleeding. Prior study found increased risk of bleeding in those with platelet counts ≤5,000 cells/μL compared to those with platelet counts ≥81,000 cells/μL, although there was otherwise no clear correlation of decreased bleeding risk with increased platelet counts (8). There is no platelet count threshold at which the risk of bleeding cannot be accounted for (9), and hemorrhagic complications directly impact survivorship among patients with malignancy (10). Thrombocytopenia often carries prohibitive surgical risk and is a relative contraindication for percutaneous pericardiocentesis (11). Traditional approach included attempts to correct thrombocytopenia with prophylactic platelet transfusion with a platelet count goal >50 × 10^3^ cells/μL (12).

Our study analyzed the prognostic factors and overall survival (OS) of pericardiocentesis in thrombocytopenic patients with diagnosis of malignancy and attempted to determine the utility of platelet count and hemostatic evaluations in predicting bleeding risk, hypocoaguable state, and mortality among thrombocytopenic cancer patients undergoing pericardiocentesis. To our knowledge, this was the first retrospective survivorship analysis of this particular patient population after primary percutaneous pericardiocentesis.

## Materials and Methods

The Institutional Review Board of The University of Texas MD Anderson Cancer Center (MDACC) approved this study with a waiver for written informed consent. In December 2018, a retrospective review of the MDACC cardiac catherization laboratory registry for cancer patients with platelet counts <150 × 10^3^ cells/μL who underwent primary percutaneous pericardiocentesis between October 1, 2009 to November 30, 2018 was performed. In total, 136 patients met the criteria above and were included in this study. Severity of thrombocytopenia was classified based on platelet count recorded for each patient on the day of pericardiocentesis based on NCI-CTCAE version 5 criteria (13): grade 1 (75–149 × 10^3^ cells/μL), grade 2 (50–74 × 10^3^ cells/μL), grade 3 (25–49 × 10^3^ cells/μL), and grade 4 (<25 × 10^3^ cells/μL). Recorded data included patient demographics, cancer history, and serological test results obtained 24 h peri-procedurally (Table 1) and echocardiographic data with evidence of increased pericardial pressure or cardiac tamponade physiology. Patients then underwent percutaneous pericardiocentesis with an indwelling pigtail catheter placement (5F Cook pericardial drain) preferably for 3–5 days. The catheter was removed if fluid drainage dropped below 25–50 mL with no residual effusion seen by follow-up echocardiography.

**Table 1:** Descriptive statistics of patient characteristics.

**Categorical variable**		***N* (%)**	**Continuous variable**	**Mean ± SD, Median (Min, Max)**	***N***
Gender	Male	82 (60.3%)	Age (years)	53.27 ± 17.68, 56.16 (17.86, 84.77)	136
	Female	54 (39.7%)	Weight (kg)	77.54 ± 19, 75.8 (43.6, 134.5)	136
Race	White	82 (60.3%)	Height (cm)	169.76 ± 13.24, 170.1 (76, 196)	136
	Hispanic	18 (13.2%)	BMI (kg/m^2^)	27.67 ± 13.42, 26.27 (16.73, 165)	136
	African American	19 (14%)	BSA (m^2^)	1.89 ± 0.25, 1.89 (1.4, 2.51)	136
	Other	17 (12.5%)	Troponin I (ng/mL)	6.36 ± 50.15, 0.03 (0, 492)	103
Cancer type	Solid	42 (30.9%)	Troponin T (ng/mL)	21.13 ± 14.56, 19.5 (6, 43)	8
	Hematologic	98 (69.1%)	BNP (pg/mL)	509.05 ± 847.69, 241 (1.49, 5,479)	101
Primary cancer	Breast	4 (2.9%)	NT-proBNP (pg/mL)	737 ± 523.03, 650 (212, 1,582)	7
	Gastrointestinal	7 (5.1%)	Serum creatinine (mg/dL)	1.23 ± 1.11, 0.94 (0.3, 10.63)	136
	Genitourinary	3 (2.2%)	WBC (cells/mL^3^)	5.51 ± 6.69, 3.55 (0, 41)	136
	Gynecologic	4 (2.9%)	Hemoglobin (g/dL)	9.48 ± 1.65, 9.1 (6.7, 14.6)	136
	Head and Neck	1 (0.7%)	pRBC administered within 24 h (units)	0.21 ± 0.49, 0 (0, 2)	23
	Leukemia	65 (47.8%)	Platelet count (day 0) (K/mL)	64.46 ± 45.07, 51 (6, 147)	136
	Lung	16 (11.8%)	Grade 1 (75–149 × 10^3^ cells/μL)		55
	Lymphoma	29 (21.3%)	Grade 2 (50–74 × 10^3^ cells/μL)		14
	Melanoma	1 (0.7%)	Grade 3 (25-49 × 10^3^ cells/μL)		33
	Renal	1 (0.7%)	Grade 4 (0–24 × 10^3^ cells/μL)		34
	Sarcoma	4 (2.9%)	Platelet administered within 24 h (units)	1.57 ± 3.42, 0 (0, 23)	37
	Thymus	1 (0.7%)	INR	1.31 ± 0.28, 1.26 (0.87, 3.05)	136
Advanced cancer		105 (77.2%)	LVEF (%) by TTE	55.23 ± 9.37, 55 (25, 70)	136
History of radiotherapy		44 (32.4%)			
Chemotherapy within 1 month		92 (67.6%)			
Tobacco smoker within 1 year		41 (30.1%)			
Hypertension		57 (41.9%)			
Dyslipidemia		87 (64%)			
Chronic lung disease		13 (9.6%)			
Diabetes mellitus		15 (11%)			
CKD, dialysis-dependent		2 (1.5%)			
Cerebrovascular disease		7 (5.1%)			
Coronary artery disease		6 (4.4%)			
Chronic heart failure		12 (12.6%)			
Family history premature CAD		8 (5.9%)			
Aspirin use only		14 (10.3%)			
Clopidogrel use only		3 (2.2%)			
DOAC use only		9 (6.6%)			
Platelet transfusion refractoriness		27 (19.9%)			
Cardiac tamponade on TTE		68 (50%)			
Complications		5 (3.7%)			
Procedural guidance modality	Echocardiogram	131 (96.3%)			
	Fluoroscopy	61 (44.9%)			
	Combined	96 (70.6%)			
Aspirated fluid appearance	Serous	57 (41.9%)			
	Hemorrhagic	79 (58.1%)			
Malignant aspirated fluid		56 (41.2%)			

*CKD, chronic kidney disease; CAD, coronary artery disease; DOAC, direct oral anticoagulant; TTE, transthoracic echocardiogram*.

Recording a successful pericardiocentesis required an accurate technique with meticulous hemostasis, equipment availability (7 and 12 cm Cook micropuncture kits), image guidance (when possible a “triple safety” approach consisting in ultrasound-guided needle advancement, fluoroscopy, and echocardiography), and proficiency in subxiphoid and apical approach. Percutaneous pericardiocentesis was performed by using the shortest distance to the pericardial cavity from the subxiphoid or intercostal space, and using the 5-F micropuncture kit (Micropuncture Introducer Kit, Silhouette Transitionless Push-Plus Design, Cook Medical, Bloomington, Indiana) in order to reduce the bleeding risk, with intercostal site entry (lateral) being the preferred approach. Based on body habitus, skin and breast anatomy, scarring from previous surgeries, mediastinal shift from underlying malignancy or abdominal distension, lateral approach expanded between 4 and 6th intercostal space and from midclavicular to midaxillary line. In procedures where only echocardiographic guidance was available, or in patients with concomitant pericardial and pleural effusion or ascites, upon accessing the pericarial space, position was confirmed with agitated saline injection, followed by advancement of micropuncture dilator and additional confirmation with “microbubbles,” and completed with the advancement of the multi-hole pigtail catheter under fluoroscopic guidance and suturing in place. In complex (unstable, challenging) patients where both echocardiographic and fluoroscopic guidance were available, to avoid incidental needle displacement and increase in procedural time, if fluid was serous, the pericardial space was secured advancing the micropuncture guidewire with fluoroscopic confirmation of the intrapericardial position prior to advancement. Fluid samples were sent to pathology and microbiology for analysis and results were documented.

Patient demographical characteristics were summarized using mean (SD) and median (minimum-maximum) for continuous variables and counts (%) for categorical variables. Overall survival (OS, time interval from procedure (pericardiocentesis) to death or last follow up) was calculated. Univariate and multivariate Cox proportional hazards regression analyses were conducted to identify variables that were associated with increased risk of death. Kaplan-Meier survival plots were generated and log-rank test was used to compare among subgroups in OS. Estimated median follow-up using reverse Kaplan-Meier method was used, considering the event of death as a censor, so that unobservable follow-up time of each subject was interpreted as follow-up time. A *p* < 0.05 indicated a statistical significance. SAS 9.4 (SAS Institute INC, Cary, NC) was used for data analysis.

## Results

**Figure 1:**
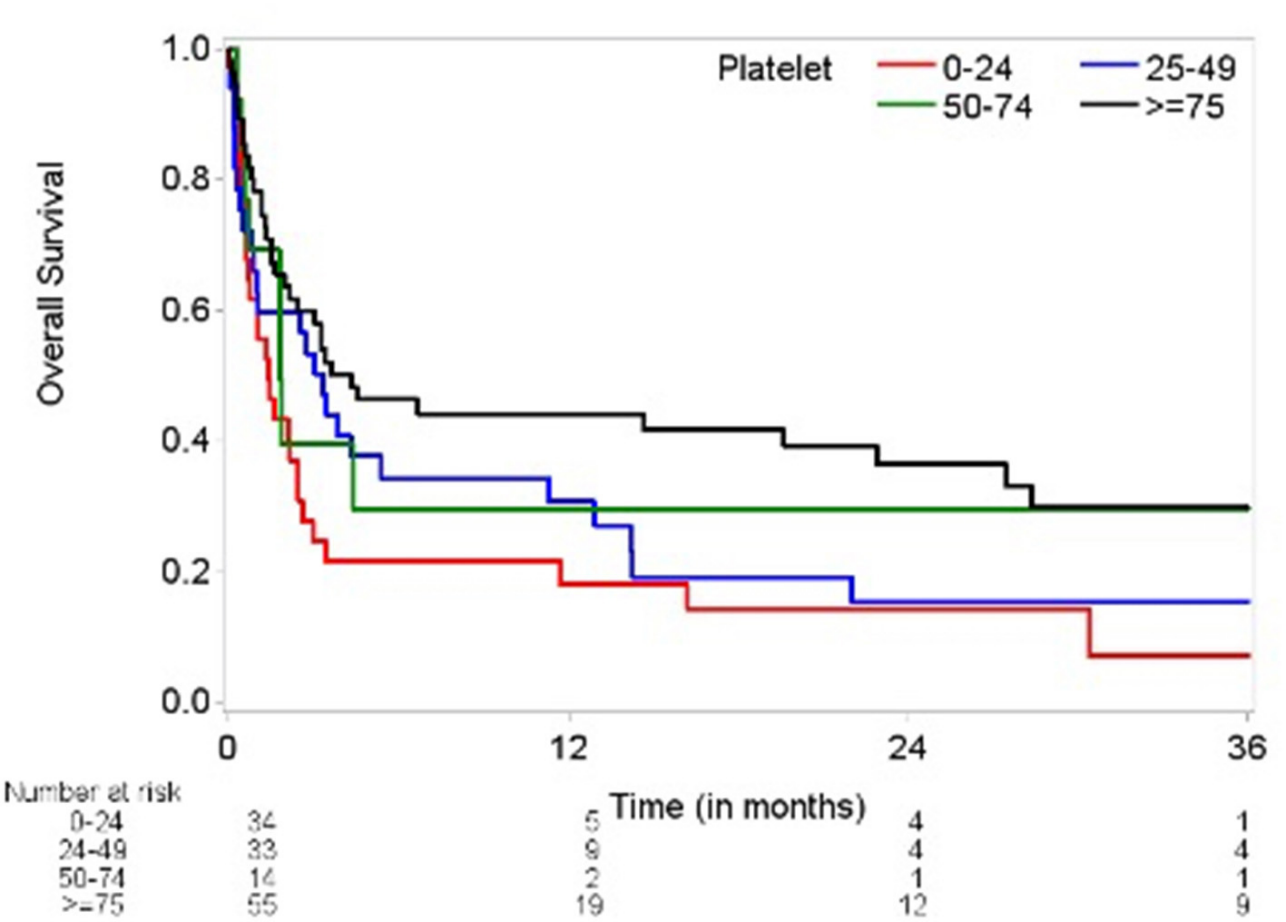
Kaplan-Meier plot of overall survival by thrombocytopenia severity (log-rank test *p* = 0.0234). Time (months) on the *x*-axis marks time elapsed from pericardiocentesis. Overall survival (percentage) on the *y*-axis. Number at risk delineates the remainder of surviving patients at each time point in each group based on degree of thrombocytopenia and platelet count.

Our study included 136 patients with malignancy stratified by grade of severity of thrombocytopenia: 41% grade 1 (75–149 × 10^3^ cells/μL); 10% grade 2 (50–74 × 10^3^ cells/μL); 24% grade 3 (25–49 × 10^3^ cells/μL); 25% grade 4 (<25 × 10^3^ cells/μL) (Tables 1, 2). Of the 136 patients, 35 survived during the follow-up period. After pericardiocentesis, median OS using reverse Kaplan-Meier method was 2.6 months with a median follow-up of 21.4 months (95% confidence interval (CI) 0.2–106.8 months). Significant OS differences were observed across thrombocytopenia grades recorded on day 0 (*p* = 0.023, Figure 1). Evaluation of patients based on platelet counts <100 × 10^3^ cells/μL or ≥100 × 10^3^ cells/μL showed a statistical significance in OS (*p* = 0.031). However, there were more patients with platelet count ≥100 × 10^3^ cells/μL without advanced cancer than with advanced cancer (54.84 vs. 22.86%, *p* = 0.0007).

**Table 2:** Univariate analysis for impact on overall survival.

**Categorical variable**		**Hazard ratio (95% CI)**	***p*-value**
Gender	Male	1	
	Female	0.838 (0.565–1.243)	0.3796
Race	White	1	
	Hispanic	0.986 (0.541–1.798)	0.964
	African American	1.067 (0.605–1.883)	0.8223
	Other	1.148 (0.616–2.136)	0.6643
Cancer type	Solid	1	
	Hematologic	0.753 (0.492–1.154)	0.1931
Primary cancer	Breast	1.704 (0.524–5.537)	0.3755
	Gastrointestinal	0.517 (0.187–1.430)	0.2038
	Genitourinary	0.815 (0.198–3.354)	0.7768
	Gynecologic	1.492 (0.463–4.811)	0.5032
	Head and Neck	2.117 (0.289–15.491)	0.4601
	Leukemia	1	
	Lung	1.471 (0.813–2.660)	0.202
	Lymphoma	0.520 (0.296–0.911)	**0.0223**
	Melanoma	0.000 (0.000)	0.9867
	Renal	6.707 (0.886–50.760)	0.0653
	Sarcoma	1.126 (0.350–3.621)	0.8419
	Thymus	0.000 (0.000)	0.9903
Advanced cancer		10.717 (4.345–26.433)	**<0.0001**
History of radiotherapy		1.351 (0.892–2.046)	0.1549
Chemotherapy within 1 month		1.538 (0.892–2.396)	0.0565
Tobacco smoker within 1 year		1.382 (0.988–2.108)	0.1336
Hypertension		0.662 (0.445–0.984)	**0.0416**
Dyslipidemia		0.791 (0.525–1.192)	0.2624
Chronic lung disease		0.938 (0.488–1.802)	0.8467
Diabetes mellitus		0.643 (0.334–1.239)	0.1869
CKD, dialysis–dependent		2.673 (0.654–10.922)	0.1709
cerebrovascular disease		0.483 (0.153–1.530)	0.2164
Coronary artery disease		0.763 (0.280–2.077)	0.5959
Chronic heart failure		1.069 (0.606–1.886)	0.8182
Family history premature CAD		0.615 (0.249–1.520)	0.2926
Aspirin use only		0.543 (0.281–1.049)	0.0691
Clopidogrel use only		0.275 (0.038–1.980)	0.2001
DOAC use only		1.023 (0.448–2.337)	0.9569
Platelet transfusion refractoriness		1.874 (1.201–2.925)	**0.0057**
Cardiac tamponade on TTE		1.269 (0.857–1.879)	0.2337
Complications		0.707 (0.224–2.232)	0.5541
Procedural guidance modality	Echocardiogram	0.634 (0.257–1.563)	0.3224
	Fluoroscopy	0.944 (0.638–1.399)	0.775
	Combined	1.108 (0.712–1.725)	0.6481
Aspirated fluid appearance	Serous	1	
	Hemorrhagic	0.814 (0.546–1.214)	0.3131
Malignant aspirated fluid		1.659 (1.117–2.465)	**0.0122**
Age (years)		1.003 (0.992–1.014)	0.6026
Weight (kg)		0.999 (0.988–1.009)	0.816
Height (cm)		0.988 (0.974–1.002)	0.1027
BMI (kg/m^2^)		1.008 (0.994–1.022)	0.2675
BSA (m^2^)		0.826 (0.368–1.855)	0.6427
Troponin I (ng/mL)		0.996 (0.988–1.005)	0.3961
Troponin T (ng/mL)		1.018 (0.959–1.081)	0.5521
BNP (pg/mL)		1.000 (1.000–1.000)	0.8847
NT–proBNP (pg/mL)		1.000 (0.998–1.002)	0.8009
Serum creatinine (mg/dL)		1.000 (0.857–1.166)	1
WBC (cells/mL^3^)		1.008 (0.975–1.042)	0.6452
Hemoglobin (g/dL)		1.015 (0.901–1.143)	0.8121
pRBC administered within 24 h (units)		1.297 (0.880–1.913)	0.0.1886
Platelet count (day 0) (K/mL)		0.993 (0.989–0.997)	**0.0021**
Grade 1 (75–149 × 10^3^ cells/μL)		1	
Grade 2 (50–74 × 10^3^ cells/μL)		1.276 (0.592–2.753)	0.5336
Grade 3 (25–49 × 10^3^ cells/μL)		1.530 (0.928–2.522)	0.0959
Grade 4 (0–24 × 10^3^ cells/μL)		2.102 (1.288–3.431)	**0.0029**
Platelet administered within 24 h (units)		1.055 (1.003–1.110)	**0.0374**
INR		2.583 (1.279–5.219)	**0.0082**
LVEF (%) by TTE		1.010 (0.987–1.033)	0.3888

*SD, standard deviation; CI, confidence interval; BMI, body mass index; BSA, body surface index; BNP, brain natriuretic peptide; NT-proBNP, N-terminal pro brain natriuretic peptide; WBC, white blood cell: pRBC, packed red blood cell INR, international normalized ratio; LVEF, left ventricular ejection fraction; TTE, transthoracic echocardiogram. Boldface indicates statistical significance*.

Variables showing significant association with OS based on univariate Cox models include elevated INR, platelet count on day of procedure, thrombocytopenia severity grade on day of procedure, platelet transfusion within 24 h of procedure, advanced cancer status, malignant fluid composition, and platelet resistance. Factors that did not show significant associations with OS included hemoglobin level, quantity of red blood cell transfusions, anticoagulant therapy, age, race, gender, cardiac tamponade, heart failure, prior radiotherapy, or recent chemotherapy.

The increased recorded platelet count as a continuous variable on procedure day was significantly associated with decreased risk of death (hazard ratio (HR) 0.993; 95% CI 0.989–0.997; *p* = 0.002). Thrombocytopenia grade 4 (HR 2.10; 95% CI 1.29–3.43; *p* = 0.003) comparing to grade 1 was associated with increased risk of death. Poor prognostic factors for OS were advanced cancer, malignant effusion, elevated INR, quantity of platelet transfusions, and platelet transfusion resistance. Adjusting for INR (HR 2.739; 95% CI 1.382–5.428; *p* = 0.004) and advanced cancer status (HR 10.865; 95% CI 4.328–27.277; *p* < 0.0001), thrombocytopenia severity grade on day of procedure (*p* = 0.802) became insignificant (Table 3). Based on the current data, the majority of patients had advanced cancer [105 (77%) with advanced cancer vs. 31 (23%) with non-advanced cancer] and the majority of patients with higher thrombocytopenia grade had advanced cancer (85–88% with advanced cancer for grades 2, 3, and 4). Including 105 patients with advanced cancer, thrombocytopenia grade was not significantly associated with OS in a univariate Cox model (*p* = 0.736) and in a multivariate Cox model (*p* = 0.887), adjusting for INR (HR 2.588; 95% CI 1.261–5.311; *p* = 0.010). Marginally significant association was observed in platelet count (as a continuous variable) in a univariate Cox model (HR 0.981; 95% CI 0.960–1.003; *p* = 0.087) and a multivariate Cox model (HR 0.980; 95% CI 0.958–1.002; *p* = 0.077), adjusting for INR (HR 65.396; 95% CI 0.986–4335.876; *p* = 0.051) including patients with non-advanced cancer. However, this multivariate Cox model included 5 events which are not large enough number of events to provide reliable HR estimates.

**Table 3:** Overall survival by multivariate analysis including INR and advanced cancer status.

**Variable**	**Level**	**Hazard ratio (95% CI)**	***p*-value**
INR	In 1-unit change	2.739 (1.382–5.428)	**0.0039**
Platelet count (day 0) (K/mL)	Grade 1 (75–149 × 10^3^ cells/μL)	1.000	
	Grade 2 (50–74 × 10^3^ cells/μL)	0.872 (0.403–1.885)	0.7270
	Grade 3 (25–49 × 10^3^ cells/μL)	0.861 (0.518–1.431)	0.5646
	Grade 4 (0–24 × 10^3^ cells/μL)	1.112 (0.667–1.855)	0.6845
Advanced cancer	Yes	10.865 (4.328–27.277)	**<0.001**

*INR, international normalized ratio. Boldface indicates statistical significance*.

Pericardiocentesis was performed via subxiphoid (16, 12%) and left apical (120, 88%) approaches. One patient with grade 1 thrombocytopenia developed a hematoma at the pericardial drain site. In addition to the hematoma, other periprocedural issues involved shoulder pain (1 patient), and transient pericarditis (3 patients). Of these 5 patients, the 3 patients with pericarditis survived past 2 months. Three out of 6 (50%) patients who died within 60 days all suffered from advanced malignancy and coagulopathy with elevated INR. Other than the one patient with hematoma, there were no significant periprocedural bleeding complications, regardless of platelet count.

Pericardial window was performed in 6 patients, four of whom survived past 1 month. Platelet counts on day 0 for patients undergoing pericardial window ranged from 12 × 10^3^ to 106 × 10^3^ cells/μL. The two patients who did not survive had additional neutropenia and one elevated international normalized ratio (INR) level. All had advanced cancer staging (4 leukemia, 1 lymphoma, 1 lung cancer) with recurrent PEs after subsequent pericardiocentesis.

Pre-procedural platelet transfusions were administered for 36 patients (26%), 27 of whom were determined to have platelet transfusion refractoriness, defined as post-transfusion platelet count increment < 10 × 10^3^ cells/μL within 24 h after platelet transfusion.

Thromboelastography (TEG) was performed in 8 patients prior to pericardiocentesis, among patients in all four grades of thrombocytopenia. TEG results revealed hypocoagulability in 4 patients (2 with grade 1, 2 with grade 3, 1 with grade 4); 3 TEGs revealed normal clotting function (2 with grade 3, 1 with grade 4), and 1 revealed hypercoagulability (grade 1) (Table 4). Five of the patients with performed TEGs presented in cardiac tamponade. Only one patient with TEG evaluation received pre-procedural platelet transfusion (patient had hypocoaguable TEG result, with grade I thrombocytopenia).

**Table 4:** Thromboelastography (TEG) interpretation, by platelet group.

**Platelet group (*n* = 8)**	**Hypocoaguable TEG**	**Normal TEG**	**Hypercoagulable TEG**
Grade 1 (platelet count 75–149 × 10^3^ cells/μL)	1		1
Grade 2 (platelet count 50–75 × 10^3^ cells/μL)			
Grade 3 (platelet count 25–49 × 10^3^ cells/μL)	2	2	
Grade 4 (platelet count 0–24 × 10^3^ cells/μL)	1	1	

*TEG, interpretation based on pathologist review*.

## Discussion

Patients with PE and underlying malignancy typically present less acutely without hemodynamic compromise, and face decreased OS compared to those without malignancy (1). The only serological marker shown to have a statistically significant negative influence on OS was elevated INR, indicating underlying coagulopathy may worsen overall prognosis. INR was elevated for several non-specific reasons in these patients, however, including anticoagulation, liver dysfunction, malnutrition, vitamin K deficiency. Patients with elevated INR tended to be very ill, and the severity of their disease likely contributed to the correlation with trends for worse OS. Similarly, advanced cancer status was heavily correlated with degree of thrombocytopenia, and likely a confounding variable for OS as highlighted in Table 3. After multivariate analysis, thrombocytopenia severity was not significantly associated with OS when advanced cancer and elevated INR were also accounted for.

Worse OS was associated with advanced carcinoma stage and malignant etiology of effusion. However, chemotherapy, radiotherapy, or concomitant infection did not show statistical significance in respective effects on OS. At 1 year post-pericardiocentesis, increased mortality was noted in patients with thrombocytopenia grades 2, 3, and 4 that also correlated with cancer severity and was attributed to natural progression of cancer.

Approach to the pericardial space has evolved; the preferred approach in thrombocytopenic patients is lateral with intercostal site entry between 4 and 6th intercostal spaces and from midclavicular to midaxillary line, unless there are adhesions between the left ventricular apex and pericardial sac or technical barriers to access (skin infections, scars from previous interventions, implants, additional pleural effusion) or the access to the pericardial space is through reduced amount of tissue and avoids hepatic structure or the loculated pocket is accessible only subxiphoid (14). A large study at our institution of pericardiocentesis in malignant PE yielded procedural site selection rates of subxiphoid approach in 63% and lateral intercostal approach in 37% of patients, with low complication rates (12).

**Figure 2:**
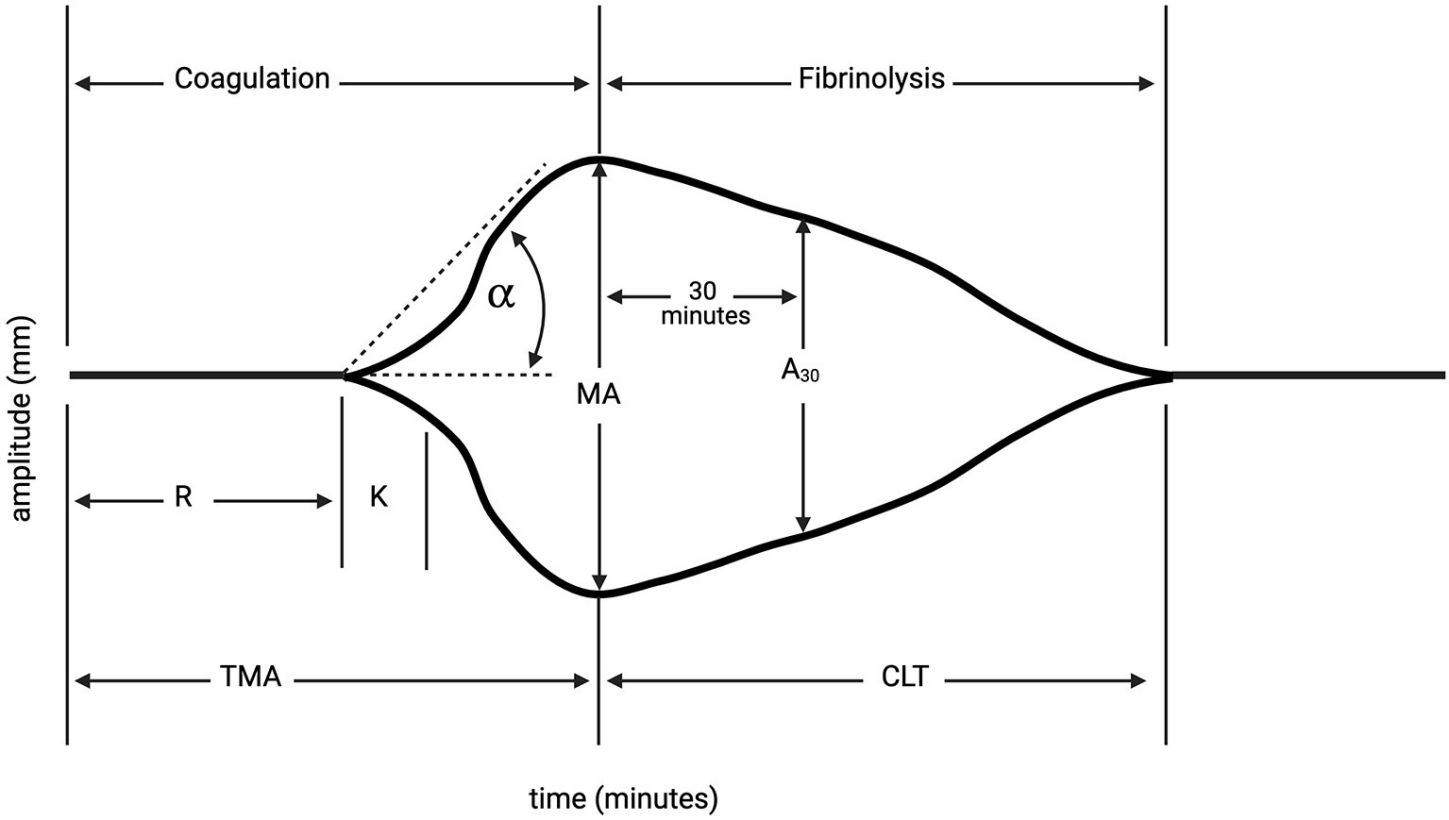
Thromboelastography (TEG). TEG is a hemostatic blood test which dynamically evaluates platelet function and clotting efficacy of whole blood. Reaction time (R) is latency from start of test to initial fibrin formation, and is dependent on clotting factors (normal: 4–8 min). Kinetics (K) is the duration taken to achieve clot strength of amplitude 20 millimeters (mm) (normal: 1–4 min). Alpha (α) measures the speed of fibrin cross-linking, dependent on fibrinogen (normal: 47–74°). Maximum amplitude measures ultimate strength and stability of fibrin clot (mm) (normal: 55–73 mm). Time to reach maximum amplitude (TMA); percentage decrease in amplitude 30-min post-MA (A_30_); Clot lysis time (CLT). At our institution, hypocoagulability on TEG is defined as a prolonged reaction time (R-time, minutes to clot formation), low alpha angle (measures clot kinetics), and low maximum amplitude (MA, millimeters, measures clot strength). Created with Biorender.com.

TEG, a hemostatic blood test which dynamically evaluates platelet function and clotting efficacy of whole blood (Figure 2), can be a helpful tool to determine bleeding risk in thrombocytopenic cancer patients prior to pericardiocentesis in stable patients (15, 16). In thrombocytopenic patients, platelet function rather than platelet count often correlates with bleeding, and hemostasis appears to be affected more than platelet adhesion (17, 18). TEG results in thrombocytopenic cancer patients with PE may provide a more comprehensive risk stratification before pericardiocentesis, and may help determine the appropriate blood product administration when hemorrhagic complications arise, approach already established for the coronary procedures (19).

In terms of intervention modality, an initial surgical approach with pericardial window could potentially provide superior results compared to percutaneous procedures due to decreased PE recurrence rates (1). When balancing the increased safety from combining echocardiographic and/or fluoroscopic guidance during percutaneous pericardiocentesis with the increased bleeding risk in thrombocytopenic patients with open surgical procedures, the clinical decision has gradually inclined toward the less-invasive approach without any apparent impact on long-term outcomes. Complication rates in this study were consistent with the low incidences reported in prior image-guided studies in non-thrombocytopenic patients, of anywhere between 4 and 20% (7, 20–23). The decreased rate of complication we assume is due to using micropuncture technique and both echocardiographic and fluoroscopic guidance, and in comlex cases even triple-guidance with additional ultrasound-guided access.

In thrombocytopenia grades 3 and 4, mortality rate only increased after 1 year and only one patient with grade 4 thrombocytopenia had peri-procedural complications. In patients with extreme thrombocytopenia we found value in having a detailed discussion with the patient and family reflecting the lack of a surgical rescue option if certain complications occur, therefore a “no plan B situations” explanation is of paramount importance before the procedure. Especially in cases of hemodynamic instability, there are no absolute contraindications for pericardiocentesis in that it may be a necessary life-saving procedure, even in patients with severe thrombocytopenia and coagulopathy (24).

**Figure 3:**
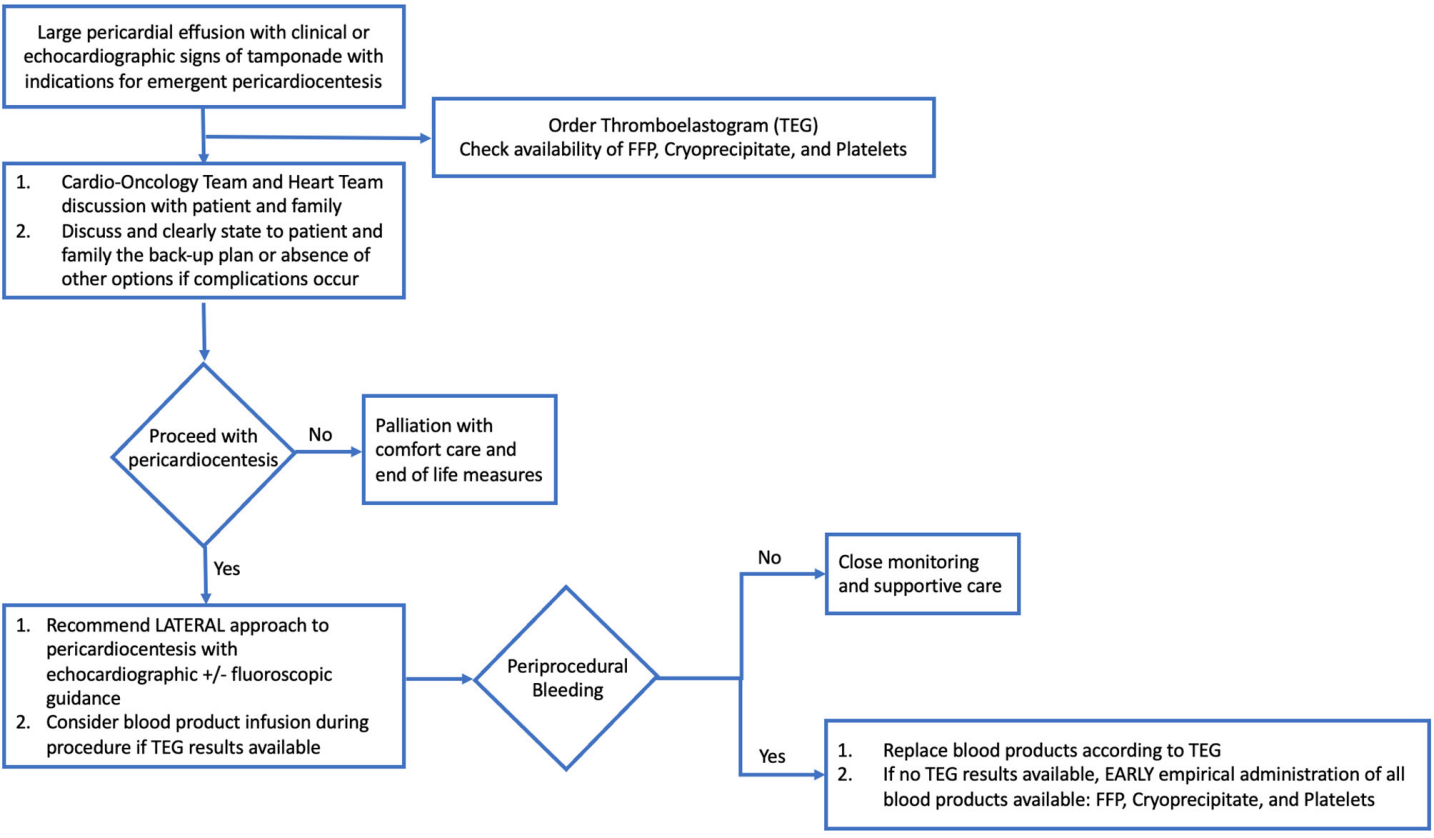
Flow-Chart: Approach to large pericardial effusion in patient with malignancy. Hemodynamically significant pericardial effusion in a patient with malignancy is a complex clinical scenario, one which requires appropriate clinical coordination, laboratory evaluation, and preparation. Important factors include a multidisciplinary discussion with Cardio-Oncology Team, Heart Team, patient and family members to discuss risks, benefits, and back-up options for complications, as well as consideration of palliation and comfort care measures. If proceeding with pericardiocentesis, recommended procedural approach is lateral and with echocardiographic ± fluoroscopic guidance, plus consideration of blood product administration based on laboratory results and thromboelastogram results. In the setting of periprocedural bleeding, early replacement of blood products according to TEG results or empiric administration of all blood products available is of paramount importance, in addition to achieving hemostasis and ongoing supportive care.

In cardiology and medicine, it is imperative to consider the ratio of risk to benefit in considering interventional diagnostic and therapeutic procedures. This study found that the greater the severity of thrombocytopenia, the greater the risk for intervention. In the case of pericardiocentesis with thrombocytopenia, the procedural risk increases as the platelet count decreases. More severe thrombocytopenia may be associated with more platelet transfusion refractoriness, less surgical back-up available, and lower overall survival. However, in patients with advanced malignancy, it is sometimes pertinent to proceed with higher risk procedures to achieve desired benefit due to the severity of disease and need for intervention. It is of utmost importance, therefore, for the cardio-oncology team to weigh risk and benefits and have the discussion with patients when performing diagnostic and therapeutic interventions such as pericardiocentesis (Figure 3). The low rate of periprocedural complications in our study may well be attributed to the consideration of these risks and benefits, and careful appropriate procedural technique.

### Limitations

Study limitations included a small sample size and the retrospective nature of data collection. A process of randomization of patients to pericardiocentesis, pericardial window, or medical therapy alone is challenging to imagine, more so to execute. Furthermore, the entry site during pericardiocentesis was ultimately dependent on patient's anatomy, ability to lie flat and interventionalists level of comfort with the approach. The use of TEG in a very small number of patients limits the ability to draw strong inferences from its interpretation. Future analysis of TEG in this patient population could be a helpful risk stratification tool. Finally, determining the utility of peri-procedural platelet transfusion is difficult since certain malignancies and their treatment can add complexity to an already coagulopathic clinical challenge.

## Conclusions

In a high-risk patient population with cancer-related large pericardial effusion and thrombocytopenia, pericardiocentesis is a feasible intervention with low complication rates when appropriate equipment and technique are used. Furthermore, there is no absolute contraindication to pericardiocentesis in cases of hemodynamic instability, even with severe thrombocytopenia. The grade of thrombocytopenia reflects disease severity; however, no significant association was observed with respect to OS when adjusting for advanced cancer status and INR. Further studies will be needed to refine the role of grade of thrombocytopenia in non-advanced cancer patients, and platelet transfusions and platelet function tests in multivariate analysis in this patient population.

## Data Availability Statement

The raw data supporting the conclusions of this article will be made available by the authors, without undue reservation.

## Ethics Statement

The studies involving human participants were reviewed and approved by The Institutional Review Board of the University of Texas MD Anderson Cancer Center. Written informed consent for participation was not required for this study in accordance with the national legislation and the institutional requirements.

## Author Contributions

NW and ML wrote the manuscript. NP, JL-M, MC, KM, and CI conceived the study, performed data analysis, and reviewed and approved the final manuscript product. NW, ML, CG, AS, TD, DB, and JS performed data collection and analysis and reviewed, edited, and approved the final manuscript product. All authors contributed to the article and approved the submitted version.

## Conflict of Interest

The authors declare that the research was conducted in the absence of any commercial or financial relationships that could be construed as a potential conflict of interest.

## Abbreviations

PE: pericardial effusion
PLADO: prophylactic platelet dose on transfusion outcomes trial
OS: overall survival
MDACC: MD Anderson Cancer Center
CI: confidence interval
CS: cumulative survival
TEG: Thromboelastography
INR: international normalized ratio
HR: hazard ratio
MA: maximum amplitude.
